# Pan-Cancer Analysis of Pentraxin 3: A Potential Biomarker of COVID-19

**DOI:** 10.3390/cancers14184438

**Published:** 2022-09-13

**Authors:** Zijian Zhou, Xuan Zhou, Yuanyuan Yang, Lujia Wang, Zhong Wu

**Affiliations:** 1 Department of Urology, Huashan Hospital, Fudan University, Shanghai 200040, China; 2 Clinical Research Center of Urolithiasis, Shanghai Medical College, Fudan University, Shanghai 200040, China; 3 Department of Urology, The First Affiliated Hospital of Nanjing Medical University, Nanjing 210029, China

**Keywords:** PTX3, pan-cancer, immunotherapy, biomarker, COVID-19

## Abstract

**Simple Summary:**

We clarified the expression patterns, genetic alterations, prognostic significance, and the underlying mechanisms of the COVID-19 biomarker PTX3 across TCGA cancers. Importantly, we linked PTX3 to immunotherapy, synthetically underscoring its importance in human cancers and providing the prospect of PTX3 and immunotherapy for tumor patients. In addition the function of PTX3 in kidney renal clear cell carcinoma (KIRC) was explored with in vitro assays. Collectively, we suggest that clinicians should pay particular attention to tumor patients with high PTX3 expression and should make specific and deliberative treatment choices.

**Abstract:**

Pentraxin 3 (PTX3), a potential biomarker of the severity and mortality of COVID-19 patients, is aberrantly expressed in human tumors. However, a comprehensive pan-cancer analysis of PTX3 remains to be elucidated. PTX3 data profiles and clinical information in TCGA cancers were obtained from different public databases to clarify the expression levels, genetic alterations, prognostic significance, underlying mechanisms, and the predicted role in immunotherapy of PTX3 across TCGA cancers. Our analyses showed that PTX3 was aberrantly expressed in most tumors and was significantly related to prognosis and tumor stage. Interaction network and enrichment analyses revealed that PTX3 participated in tumor immuno-related progression. In addition, PTX3 levels were critically associated with immune cell components and immune scores, and PTX3 strongly coexpressed with immune-related genes in TCGA cancers. Meanwhile, PTX3 expression was associated with immune checkpoint genes, and immunotherapy potential biomarkers in multiple cancers, predicting special immunotherapy responses in different tumor types. In kidney renal clear cell carcinoma (KIRC), PTX3 emerged as an independent prognostic factor through multivariable Cox regression analyses. Blocking PTX3 with siRNA could suppress the growth of KIRC cells and invasion. Conclusively, our study shows a comprehensive bioinformatic analysis of PTX3, which might serve as a pan-cancer prognostic biomarker.

## 1. Introduction

Pentraxin 3 (PTX3) is a prototypic member of the long-pentraxin subfamily [1]. As an activator of the complement pathway of the innate immune system, PTX3 is a key homeostatic component in the immune response, tissue remodeling, and cancers [2]. In recent years, PTX3 was been associated with infections, severe inflammatory response syndrome, sepsis, and cardiovascular diseases, associated with disease severity and mortality [3,4,5]. Furthermore, studies have shown that PTX3 might be involved in tumor biology and might exert distinct functions in different cancers [6,7]. PTX3 has been reported to be associated with the susceptibility to mesenchymal and epithelial carcinogenesis, macrophage infiltration, cytokine production, angiogenesis, etc. [6]. Correlative evidence has indicated that PTX3 expression was epigenetically regulated in selected human tumors by methylation of the promoter region and of a putative enhancer [8]. It has been reported that PTX3 could act as a prognostic and diagnostic marker in diffuse large B-cell lymphoma [9], ovarian epithelial cancer [10], hepatocellular carcinoma [11], etc. [12,13]. 

Recent studies have found that PTX3 was positively associated with the disease severity and mortality of severe COVID-19 patients [14,15,16]. PTX3 plasma level could help to identify severer patients on admission and might serve as an independent prognostic predictor of short-term mortality in COVID-19 [17]. Brunetta et al. found that PTX3 emerged as a strong independent predictor of 28-day mortality in a multivariable analysis, better than conventional markers of inflammation, in hospitalized patients with COVID-19 [18]. Presumably, PTX3 may be at a crossroad between COVID-19 and cancer, providing novel therapeutic opportunities for cancer patients with COVID-19. However, thus far, a systematic pan-cancer analysis of PTX3 remains to be uncharacterized. 

We conducted a bioinformatics analysis and fundamental experiments to investigate the specific and paramount roles of PTX3 in cancers. First, we found that PTX3 showed abnormal expression and correlated with the prognosis of tumor patients. Then, we examined how PTX3 regulated gene interactions and signal pathways. Noteworthy, we found that PTX3 was associated with the tumor microenvironment (TME) and immune checkpoint inhibitors (ICIs), and we researched its regulation in immunotherapy. Finally, we validated PTX3’s functions via laboratory experiments in KIRC. Our findings suggest that PTX3 might serve as a promising biomarker for predicting responses to immunotherapy and the prognoses of patients with KIRC.

## 2. Materials and Methods

### 2.1. PTX3 Data Acquisition and Processing 

PTX3 mRNA expression data and the clinicopathological information on 33 cancer types were downloaded from TCGA (https://portal.gdc.cancer.gov/, accessed on 1 April 2022), GTEx datasets (https://www.gtexportal.org/, accessed on 1 April 2022), and the GEO database (http://www.ncbi.nih.gov/geo/, accessed on 1 April 2022). Transcripts per million (TPM) were used and normalized by log2 transformation with the same sequencing platform and library preparation to minimize batch effects. The PTX3 protein profiles were obtained from the HPA (https://www.proteinatlas.org/, accessed on 1 April 2022) and UALCAN (http://ualcan.path.uab.edu/, accessed on 1 April 2022) databases. The IHC images of PTX3 were obtained from the HPA database (antibody HPA069320). The analysis of the PTX3 genomic alterations among TCGA data was calculated in the cBioPortal database (http://www.cbioportal.org/, accessed on 1 April 2022).

### 2.2. Cox Regression Analysis and Survival Analysis

Kaplan–Meier curves and univariate Cox regression analysis were used to explore the association between PTX3 expression and the prognosis of tumor patients. PTX3 expression median values were used to categorize patients into high and low expression groups. We calculated the log-rank *p*-value and Cox *p*-value with a hazard ratio (HR) for PTX3 to assess its prognostic value, including overall survival (OS) and disease-specific survival (DSS). Per endpoint, the following events were defined: for OS, death as a result of any cause; for DSS, death as a result of the target cancer and patients who died as a result of causes not related to the particular cancer were censored at their date of death. Accordingly, based on the GEO database, the prognostic value of PTX3 was confirmed in PrognoScan (http://dna00.bio.kyutech.ac.jp/PrognoScan/index.html/, accessed on 1 April 2022).

### 2.3. Protein–Protein Interaction (PPI) Network and Functional Enrichment Analysis 

The PPI network of PTX3 was established via applying the STRING database (https://string-db.org/, accessed on 1 April 2022) with the following input parameters: “full STRING network, both functional and physical protein associations”, “evidence”, and 0.400 confidence level. With the GeneMANIA (http://www.genemania.org/, accessed on 1 April 2022) database, physical interactions and coexpression were mainly picked with automatically selected weighting methods by default. 

Gene Ontology (GO) and Kyoto Encyclopedia of Genes and Genomes (KEGG) analyses were applied to research PTX3’s functions and pathways. Two sets (STRING and GeneMANIA) of genes (including C1QA, CFH, CFHR1, FCN1, FCN2, FGF2, MBL2, PTX3, APCS, NPTXR, NFKB1, TP53, IL6, FGF18, ETV6, FGF7, NPTX2, FGF20, CRP, COLEC10, RELA, FGF22, FGF16, FGF17, NCOR2, NPTX1, PTX4, and C3) were combined to perform the GO and KEGG enrichment analyses. The R package “clusterProfiler” was used to obtain the results of gene set enrichment in the human species; *p* < 0.05 and FDR < 0.20 were considered to be statistically significant. The gene set enrichment analysis (GSEA) was processed with the molecular signatures database (MSigDB) H (Hallmark gene sets) and KEGG subsets of canonical pathways and cancer modules. The GSEA results were shown using normalized enrichment scores (NES), accounting for the size and degree to which a gene set was overrepresented at the top or bottom of the ranked list of genes. The top Hallmark and KEGG terms of GSEA were exhibited with nominal *p*-value <0.05 and FDR ≤ 0.25 using the “clusterProfiler” R package.

### 2.4. Immune Analysis 

The ESTIMATE algorithm was applied to explore the level of estimated stromal and immune cells via calculating the StromalScore and ImmuneScore. Then, the infiltration levels of immune cells were assessed using the “GSVA” R package with Spearman correlations through ssGSEA. Spearman correlations were also used to generate correlation heatmaps of PTX3 with immunomodulators (immune stimulators, MHC genes, chemokines, and chemokines receptors), and the *p*-values were presented without adjustments.

The response to immunotherapy was predicted using ICIs, as well as TMB, MSI, and NEO scores. Eight relevant immune checkpoint genes were selected and their expression values were extracted. R packages “ggplot2” and “heatmap” were used. The TMB, MSI, and NEO scores were available from the UCSC XENA database and their association with PTX3 expression was explored through a Spearman correlation analysis. The TMB, MSI, and NEO scores of each patient were calculated as the observed count of somatic mutations. A Spearman analysis was used for statistical analysis, while R package “ggplot2” and “maptools” were used for visualization. 

### 2.5. Correlation of PTX3 Expression with DNA Methylation

PTX3 methylation data from the cBioPortal database were used. An analysis of the correlation between PTX3 expression and gene promoter methylation was conducted for each tumor. Correlations of PTX3 methylation with prognosis were conducted using a KM survival analysis, including OS and DSS (*p* < 0.05 as significant).

### 2.6. Cell Culture, Cell Transfection, and Quantitative Real-Time PCR (qRT-PCR)

The KIRC cell lines 786-O and Caki-1 were all obtained from the Chinese Academy of Sciences (Shanghai, China), and cultured in corresponding culture media with 10% fetal bovine serum (FBS) and 1% penicillin/streptomycin (786-O: RPMI 1640 and Caki-1: McCoy’s 5A, Gibco, Thermo Fisher Scientific, Grand Islan, NE, USA). PTX3 siRNA (HIPPOBIO Biotechnology, Nanjing, China) was transfected in the 786-O and Caki-1 cells, according to the manufacturer’s instructions. Briefly, the 786-O and Caki-1 cells were plated into each well and transfected with 10 nM siRNA in 6-well plates. After transfection for 48 h of siRNA against PTX3, total RNA was extracted using a FastPure Cell/Tissue Total RNA Isolation Kit V2 (RC112-01, Vazyme, Nanjing, China). RNA was reverse-transcribed using the HiScript III All-in-One RT SuperMix Perfect for qPCR (R333-01, Vazyme, China). The relative gene expression levels were analyzed by using qRT-PCR with the ChamQ Universal SYBR qPCR Master Mix (Q311-02, Vazyme, China). PTX3 expression was detected by qRT-PCR with three independent experiments. The reagents and consumables are listed in Supplementary Table S1. 

### 2.7. Cell Proliferation, Colony Formation Assays, and Transwell Cell Migration and Invasion Assay

First, 96-well plates were used to cultivate the 786-O (1000 cells/well) and Caki-1 (1500 cells/well) cells. After incubation at 37 °C for 1 h, cell proliferation was measured at 450 nm after 0, 24, 48, and 72 h, using a cell counting Kit-8 (CCK-8, A311-01, Vazyme, China). Transfected cells were seeded into 6-well plates at a density of 1000 per well to perform colony formation assays. After incubating for about 10 days, the colonies were fixed with 4% paraformaldehyde. After staining with crystal violet, the formed spheres were counted and photographed. Regarding invasion and migration assays, 24-well transwell inserts were coated with Matrigel, and then KIRC cells were further resuspended and seeded into the upper chambers with a serum-free medium. The full medium supplemented with 10% FBS was placed in the bottom chamber. Following incubation at 37 °C for 24 h, the cells were fixed with 4% paraformaldehyde and stained with crystal violet staining solution for 20 min. 

### 2.8. Statistical Analysis 

The statistical analysis was performed in R software (version 4.0.1) and GraphPad Prism (version 8.0). Briefly, Spearman correlation was used to test the correlations between PTX3 mRNA expression and stromal scores, immune scores, and immune regulators expression. The rational statistical test was employed to compare two independent test series (Student’s *t*-test) or more test series (ANOVA test) followed by Bonferroni’s test. Multiple comparisons were adjusted with the number of false positives method to control the familywise error rate at α = 0.05. Data were shown as average values ± standard error of the mean (SEM) and a two-sided *p* < 0.05 indicated statistical significant.

## 3. Results

### 3.1. PTX3 Expression Pan-Cancer Analysis

**Figure 1:**
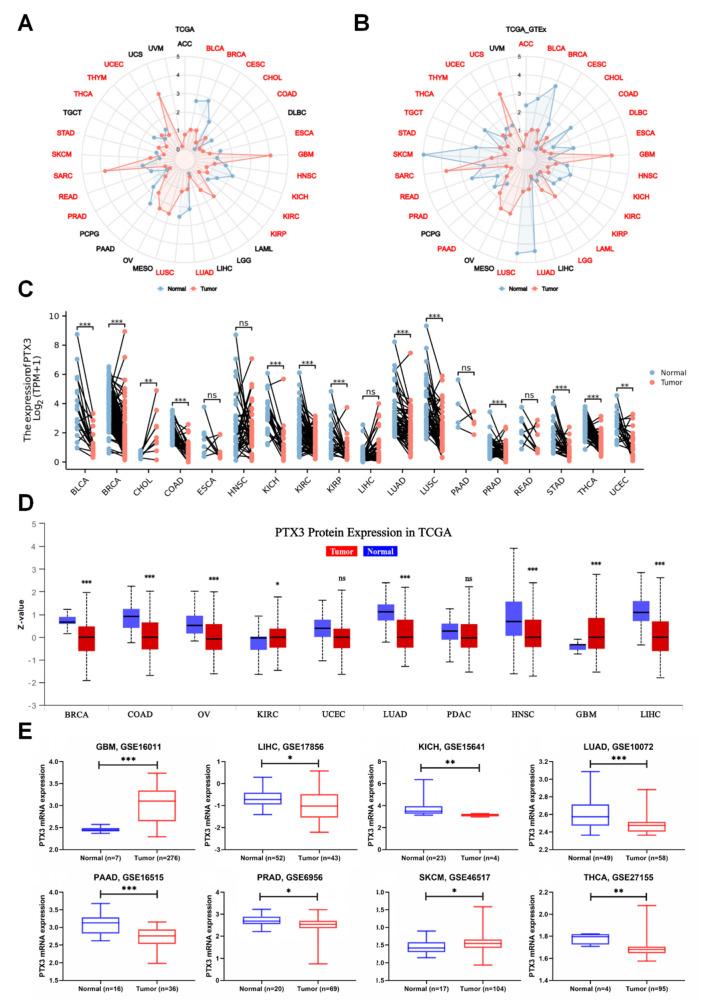
PTX3 expression in pan-cancers: (**A**) PTX3 mRNA expression in pan-cancers with TCGA (red color indicated statistical significance); (**B**) PTX3 mRNA expression in pan-cancers with GTEx_TCGA; (**C**) PTX3 mRNA expression in adjacent normal and tumor tissues; (**D**) PTX3 protein expression in pan-cancers with UALCAN; (**E**) PTX3 mRNA expression in pan-cancers with the GEO database. (ns, no significance; * *p* < 0.05, ** *p* < 0.01, *** *p* < 0.001).

**Figure 2:**
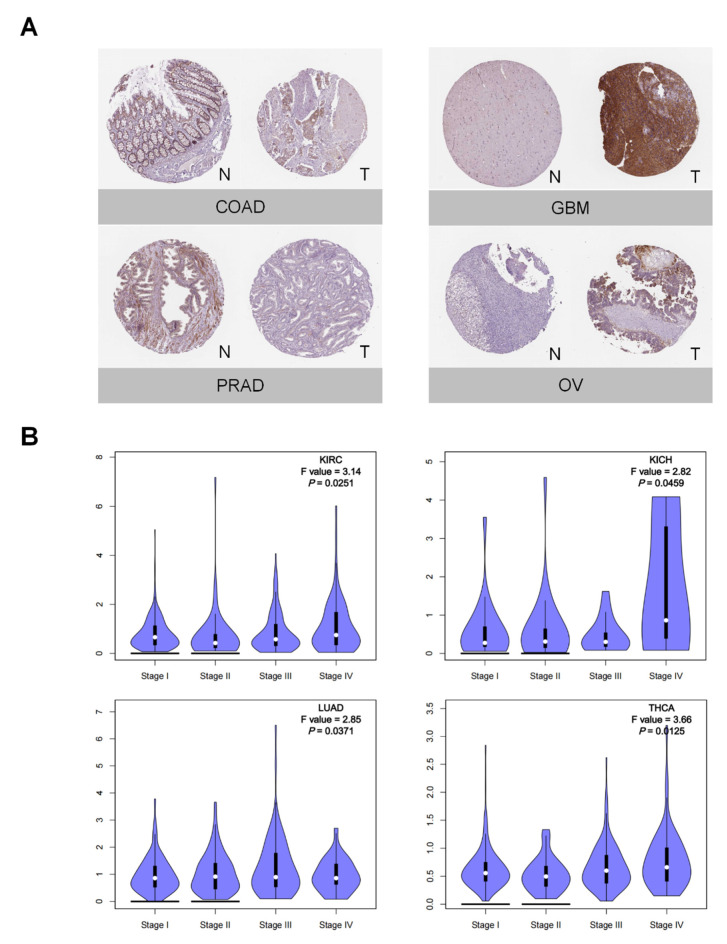
PTX3 protein expression and clinical features: (**A**) Representative immunohistochemical staining (IHC) images showed PTX3 protein expression in COAD, GBM, OV, and PRAD based on the HPA database (N, normal and T, tumor); (**B**) correlations between PTX3 and tumor stages in KIRC, KICH, LUAD, and THCA.

First, PTX3 mRNA expression was assessed. PTX3 expression was upregulated in five tumors, while it was downregulated in sixteen tumors across TCGA (Figure 1A). Overexpression of PTX3 was discovered in CHOL, GBM, SARC, SKCM, and THYM. In addition to the five previous tumors, PTX3 expression also increased in LGG, PAAD, TGCT, and UCS when the analysis was combined with the GTEx database (Figure 1B). Consistently, PTX3 mRNA expression was low in the paired pan-cancer samples except for CHOL (Figure 1C). Interestingly, with the UALCAN database, PTX3 protein expression was increased in KIRC and GBM, while it was decreased in other cancers (Figure 1D). Likewise, with the GEO database, PTX3 mRNA expression was elevated in GBM and SKCM, while it was downregulated in LIHC, KICH, LUAD, PAAD, PRAD, and THCA (Figure 1E). Then, with IHC images from the HPA database, PTX3 stronger staining was detected in GBM and OV, while PTX3 weaker staining was identified in COAD and PRAD (Figure 2A).

### 3.2. Clinical and Prognostic Significance of PTX3 in Cancers

Furthermore, PTX3 expression was correlated with tumor stage in KICH, KIRC, LUAD, and THCA (Figure 2B). The ROC analysis showed that PTX3 achieved good prediction efficacy to distinguish tumor from normal (AUC > 0.80) for BRCA, CHOL, COAD, KICH, LUSC, and THCA (Supplementary Figure S1).

**Figure 3:**
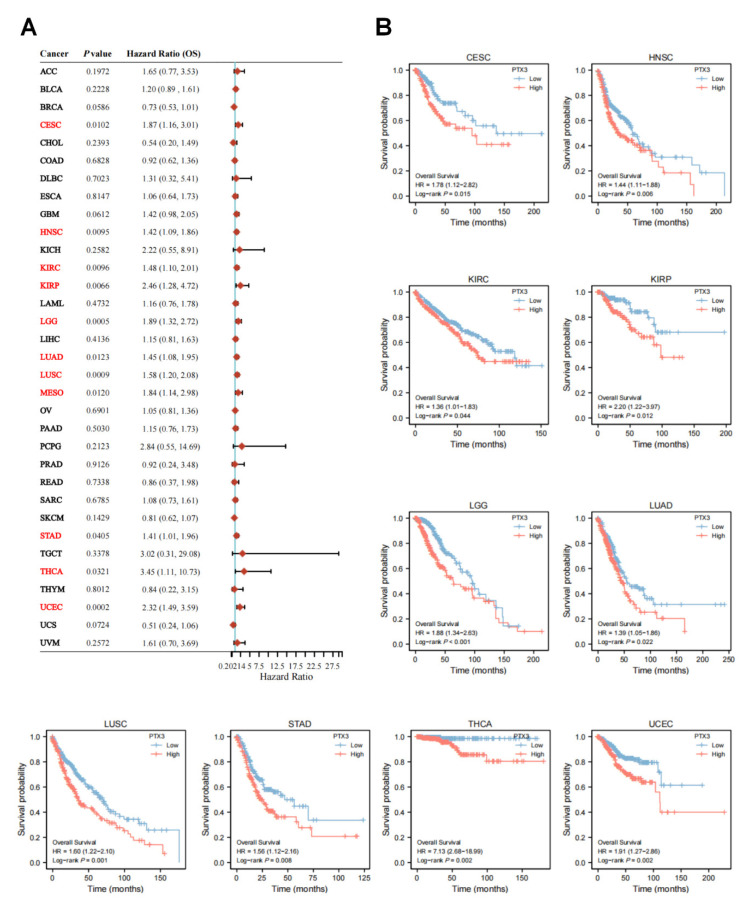
PTX3 expression with OS: (**A**) Forest plot showed univariate Cox regression analysis of PTX3 in pan-cancers (red color indicated statistical significance); (**B**) KM curves of the association between PTX3 expression and OS in cancers.

High PTX3 expression was significantly associated with poor OS in CESC (HR = 1.87, *p* = 0.0102), HNSC (HR = 1.42, *p* = 0.0095), KIRC (HR = 1.48, *p* = 0.0096), KIRP (HR = 2.46, *p* = 0.0066), LGG (HR = 1.89, *p* = 0.0005), LUAD (HR = 1.45, *p* = 0.0123), LUSC (HR = 1.58, *p* = 0.0009), MESO (HR = 1.84, *p* = 0.0120), STAD (HR = 1.41, *p* = 0.0405), THCA (HR = 3.45, *p* = 0.0321), and UCEC (HR = 2.32, *p* = 0.0002) (Figure 3A). The Kaplan–Meier (KM) OS curves obtained similar findings of the prognostic value of PTX3 (Figure 3B). 

**Figure 4:**
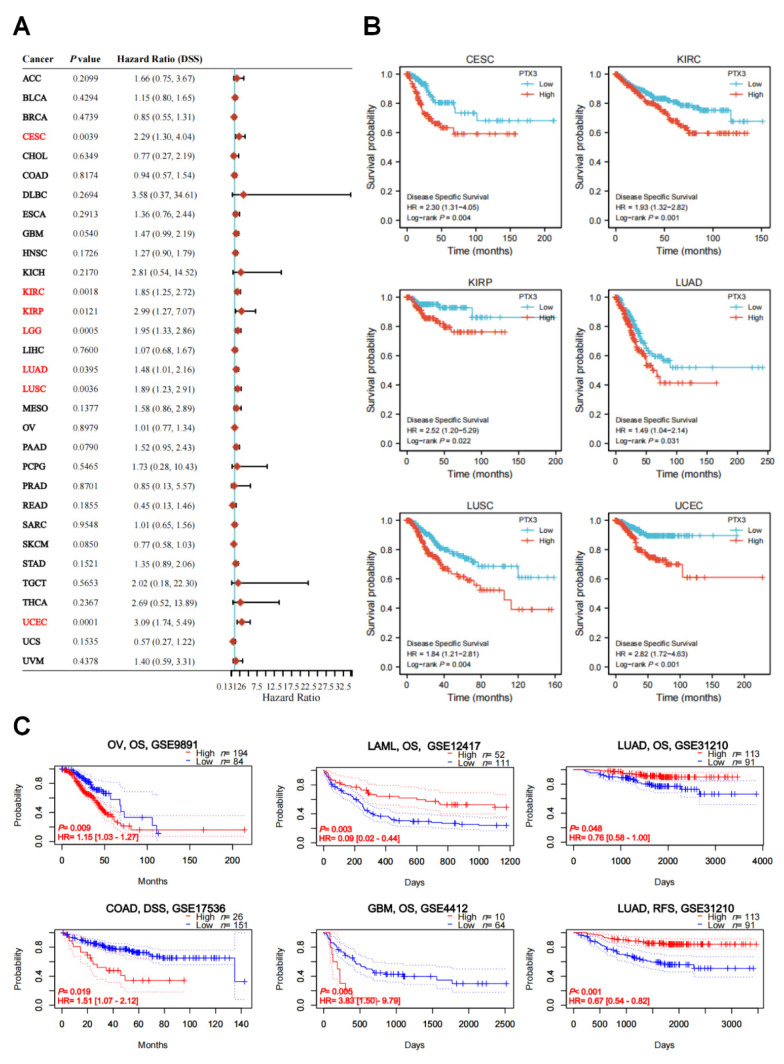
PTX3 expression with DSS: (**A**) Forest plot showed univariate Cox regression analysis of PTX3 in pan-cancers (red color indicated statistical significance); (**B**) KM curves of the association between PTX3 expression and DSS in cancers; (**C**) PTX3 prognostic value in the GEO database based on the PrognoScan database.

Figure 4A illustrated the contribution of PTX3 to DSS survival in seven cancer types: CESC (HR = 2.29, *p* = 0.0039), KIRC (HR = 1.85, *p* = 0.0018), KIRP (HR = 2.99, *p* = 0.0121), LGG (HR = 1.95, *p* = 0.0005), LUAD (HR = 1.48, *p* = 0.0395), LUSC (HR = 1.89, *p* = 0.0036), and UCEC (HR = 3.09, *p* = 0.0001). Coherently, the KM DSS curves obtained similar findings of the prognostic value of PTX3 (Figure 4B). In the GEO database, the KM curves suggested that PTX predicted poor prognoses in COAD, GBM, LAML, and OV but represented a good outcome in LUAD (Figure 4C).

### 3.3. Genetic and DNA Methylation Analysis of PTX3

The genetic alterations analysis depicted that the most frequent DNA alteration of PTX3 was amplification, which was mainly distributed in LUSC and ESCA (Supplementary Figure S2A). Most cancer patients had CNV gains and amplifications, with some deletions (Supplementary Figure S2B). Supplementary Figure S2C depicted the genomic alterations in PTX3, including their locations, types, and numbers. We detected, altogether, 47 mutation sites including 44 missenses, one truncating, one inframe, and one splice mutation between amino acids 0 and 381. The main genetic changes identified in the PTX3 gene were missense mutations. The R188C/H alteration in the pentaxin domain was detected, which was detected in one case of UCEC and one case of CESC. Importantly, PTX3 CNV was associated with poor prognosis in LGG, KIRP, PAAD, SARC, THYM, UCEC, and UVM (Supplementary Figure S3).

**Figure 5:**
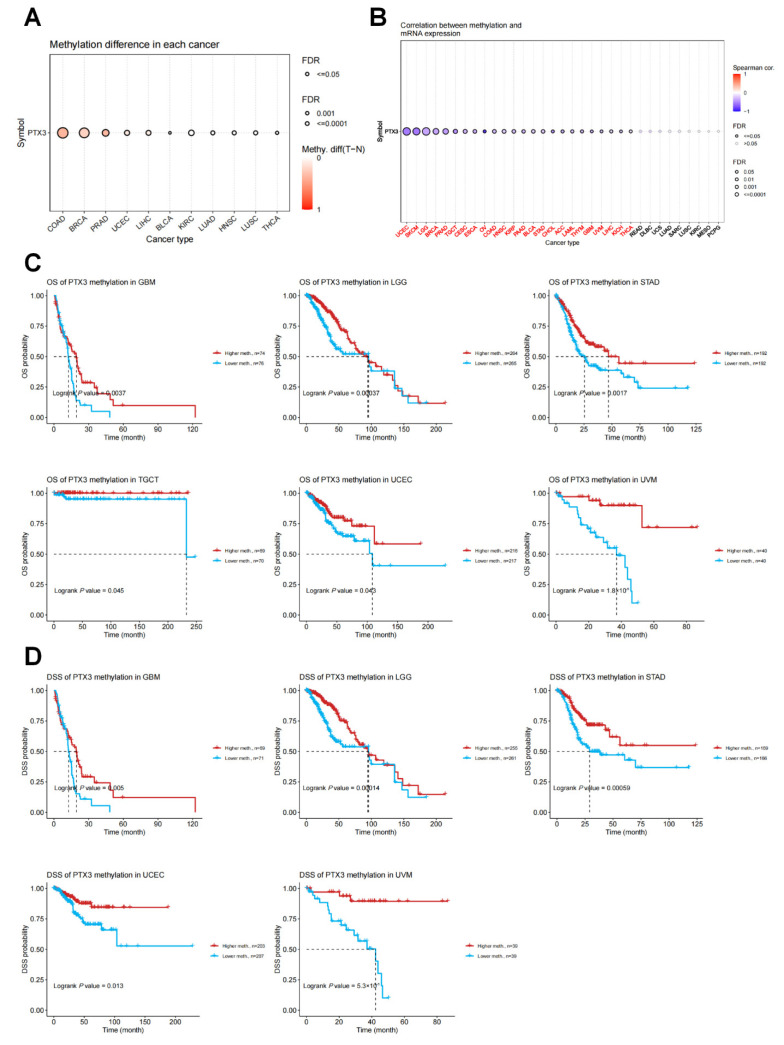
Methylation analysis of PTX3: (**A**) PTX3 methylation differences in pan-cancers; (**B**) the correlations between PTX3 expression and methylation in pan-cancers; (**C**) KM curves of the association between PTX3 promoter methylation and OS in cancers; (**D**) KM curves of the association between PTX3 promoter methylation and DSS in cancers.

Furthermore, we calculated the levels of correlation between PTX3 promoter methylation using the cBioPortal dataset and found that the methylation levels of PTX3 were lower in para-cancerous normal tissues than in tumor tissues of BLCA, BRCA, COAD, HNSC, KIRC, LIHC, LUAD, LUSC, PRAD, THCA, and UCEC (Figure 5A). We also identified significant correlations between PTX3 expression and methylation in 24 tumors, which were all negative correlations between PTX3 expression and promoter methylation levels (Figure 5B). Further, we conducted a KM survival analysis to research the relationship between PTX3 promoter methylation and patient prognosis. The PTX3 methylation level was a protective factor in patients with GBM, LGG, STAD, TGCT, UCEC, and UVM in terms of OS (Figure 5C). Regarding DSS, PTX3 methylation was a protective factor in patients with GBM, LGG, STAD, UCEC, and UVM (Figure 5D).

### 3.4. Network of PTX3 Interacting Genes

**Figure 6:**
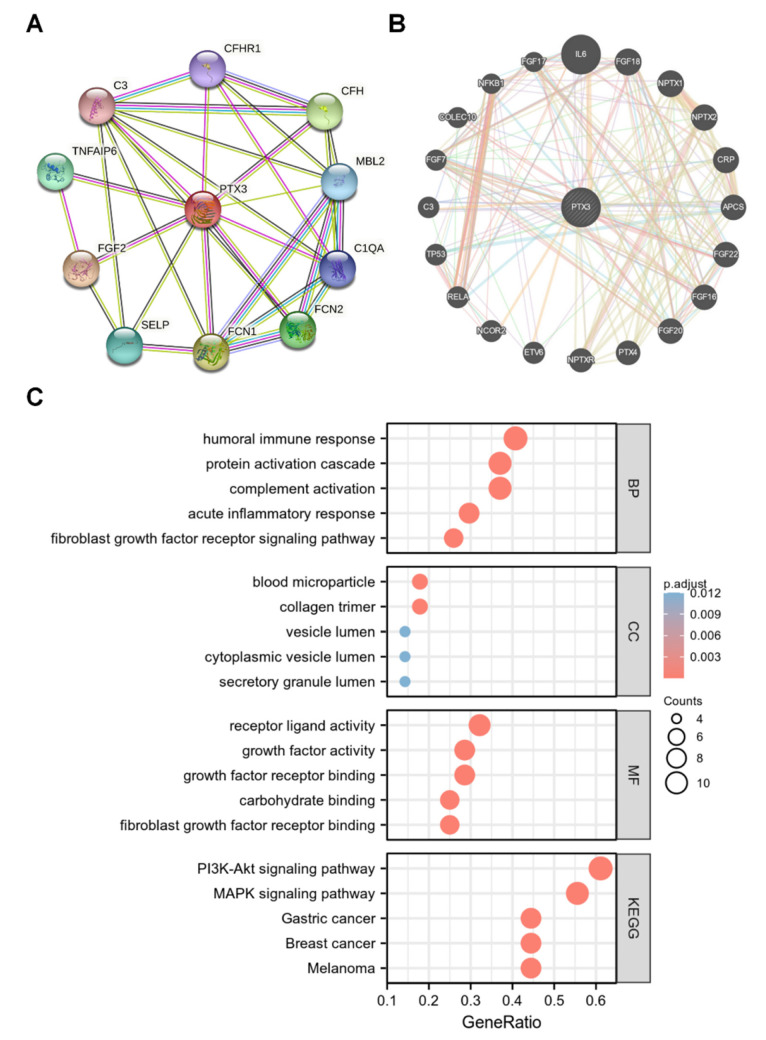
The interaction network of PTX3: (**A**) The interaction genes of PTX3 in STRING; (**B**) the PPI network of PTX3 in GeneMania; (**C**) KEGG and GO analyses of PTX3 based on the interacted genes obtained from STRING and GeneMania.

PTX3 interacting genes were investigated with the STRING and GeneMania databases. As shown in Figure 6A, PTX3 strongly interacted with C3, CFHR1, CFH, FCN1, FCN2, FGF2, MBL2, SELP, C1QA, and TNFAIP6 in the STRING database. The results of GeneMania presented interactions between PTX3 and the top 20 potential genes, including APCS, NPTXR, NFKB1, TP53, C3, IL6, etc. (Figure 6B). 

Next, GO and KEGG analyses were performed using cooperated genes in GeneMania and STRING **(**Figure 6C). Signaling pathways such as “complement activation”, “humoral immune response”, and “acute inflammatory response” were enriched in the GO analysis, while “PI3K-AKT signaling pathway”, and “MAPK signaling pathway” were enriched in the KEGG analysis, indicating that PTX3 may be involved in tumor immune response. 

### 3.5. Enrichment Analysis of PTX3

Utilizing functional KEGG and Hallmark terms through GSEA, we explored the biological mechanisms of PTX3 in pan-cancers. The top four positively enriched Hallmark terms in the high PTX3 subgroup were “fatty acid metabolism”, “estrogen response”, “pancreas beta cells”, and “oxidative phosphorylation”, while the top three negative terms included “inflammatory response”, “complement”, and “KRAS signaling” (Supplementary Figure S4A). The top three positively enriched KEGG terms included “arginine and proline metabolism”, “ribosome”, “ascorbate and aldarate metabolism”, while the top three negative terms were “focal adhesion”, “viral myocarditis”, and “Toll-like receptor signaling pathway” (Supplementary Figure S4B). These results hint that PTX3 appears to engage in the tumor immune microenvironment (TIME) and metabolism microenvironment. 

### 3.6. Immune Analysis

**Figure 7:**
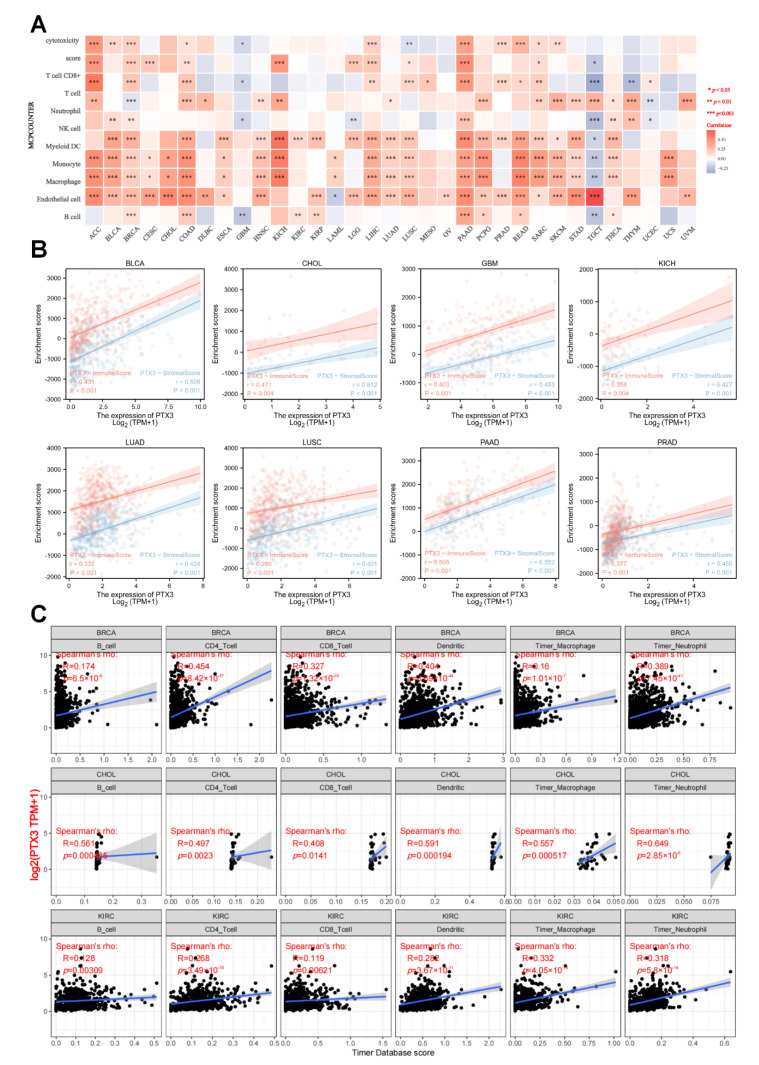
Immune analysis of PTX3: (**A**) Correlations between PTX3 and immune cells; (**B**) correlation between PTX3 and immune and stromal scores; (**C**) relationship between PTX3 and immune infiltration in the top three most related cancers.

Notably, the analysis of immune cell components exhibited that PTX3 was positively associated with most immune cells in most cancers, except TGCT (Figure 7A). At the same time, PTX3 expression was positively correlated with immune scores and stromal scores in TCGA cancers, including BLCA, CHOL, GBM, KICH, LUAD, LUSC, PAAD, and PRAD (all *p* < 0.001, Figure 7B). Meanwhile, with the TIMER database, we found that PTX3 was most positively related to immune cell infiltration in BRCA, CHOL, and KIRC (Figure 7C).

**Figure 8:**
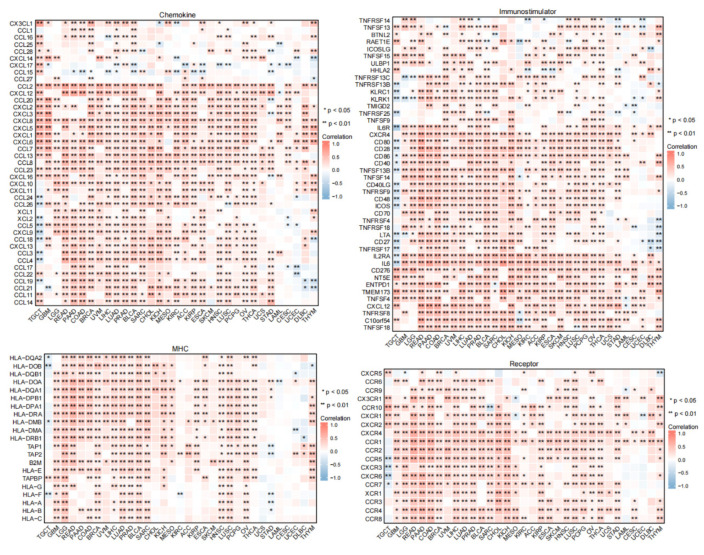
Correlation between PTX3 and immune-related genes. (Genes encoding MHC, immune activation, chemokine, and chemokine receptor proteins were analyze. * *p* < 0.05; ** *p* < 0.01).

It was shown that PTX3 expression was positively correlated with most chemokines, chemokine receptors, major histocompatibility complex (MHC) genes, and immunostimulatory genes in most human cancers, except TGCT (Figure 8). Multiple tumors showed distinct positive correlations between PTX3 expression and HLA-I and II mRNAs. In addition, among these immunostimulatory markers, a significant positive correlation was observed between PTX3 and CD276, NT5E, TMEM173, and ENTPD1. Furthermore, PTX3 expression was positively correlated with chemokines and chemokine receptors. These profiles demonstrate that PTX3 may contribute to immune-oncological interactions in TIME.

### 3.7. Predicted Role of PTX3 in Immunotherapy

**Figure 9:**
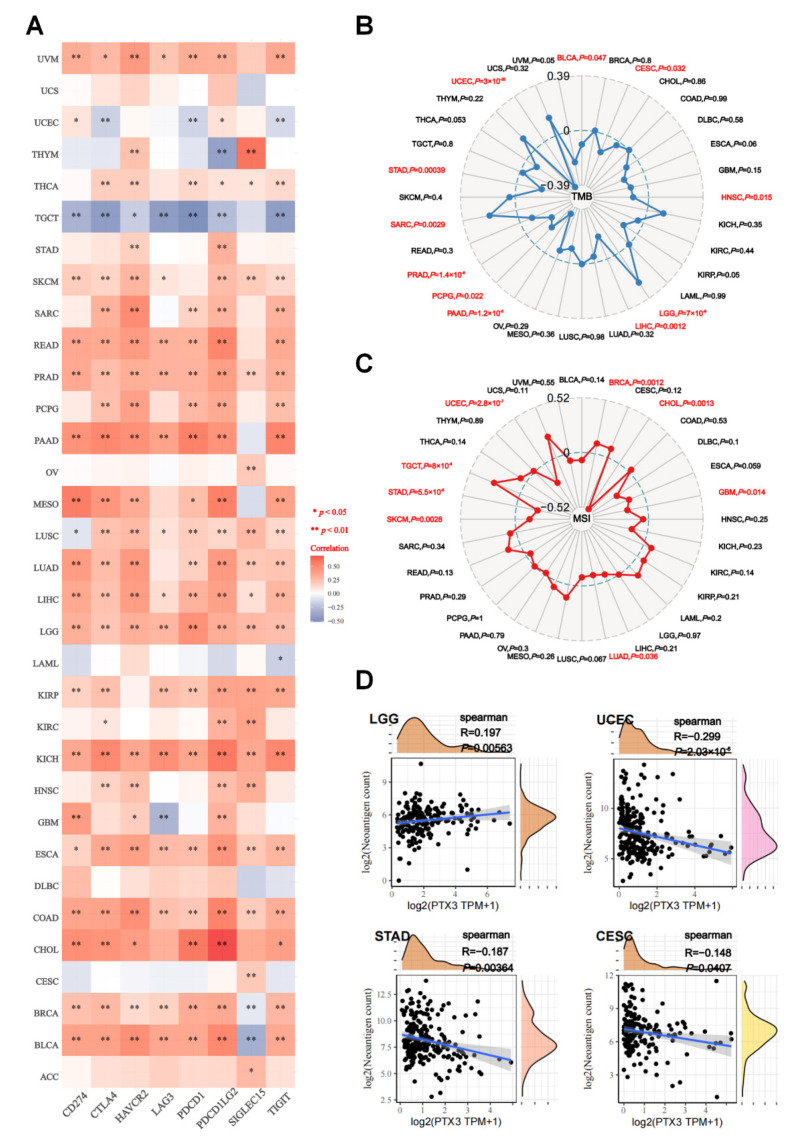
Correlation between PTX3 and immunotherapy: (**A**) Correlation of PTX3 with ICI; (**B**) radar map illustrating the relationship between PTX3 expression with TMB (red color indicated statistical significance and the red lines represent correlation coefficients); (**C**) radar map illustrating the relationship between PTX3 expression with MSI (red color indicated statistical significance and the blue lines represent correlation coefficients); (**D**) correlation of PTX3 with NEO. (Spearman correlation test, * *p* < 0.05 and ** *p* < 0.01).

ICI expressions, as well as TMB, MSI, and NEO scores have predictive values for immunotherapy [19,20]. Our findings brought to light that PTX3 had a close and positive association with most ICIs (CD274, CTLA4, HAVCR2, LAG3, PDCD1, PDCD1LG2, TIGIT, and SIGLEC15) in TCGA cancers, except TGCT (Figure 9A). In addition, PTX3 was positively correlated with TMB in LGG and SARC, while it was negatively associated with TMB in nine cancers, including BLCA, CESC, HNSC, LIHC, PAAD, PCPG, PRAD, STAD, and UCEC (Figure 9B). Additionally, negative correlations between PTX3 and MSI achieved significance in CHOL, GBM, LUAD, SKCM, STAD, and UCEC (Figure 9C). Three TCGA cohorts (CESC, STAD, and UCEC) had negative associations with NEO, while LGG had a reverse association (Figure 9D). Our results imply that PTX3 is associated with ICIs, TMB, MSI, or NEO in different types of tumors. 

### 3.8. Validation of PTX3 Function in KIRC

The bioinformatic analysis showed that PTX3 played a crucial role in human cancers including KIRC. Therefore, as urologists, we further explored the PTX3 function in KIRC. In KIRC patients, PTX3 expression was correlated with both age (*p* = 0.023) and N stage (*p* = 0.041) according to the logistic regression analysis (Table 1). The multivariable Cox regression described that PTX3 was an independent prognostic factor of OS (HR = 1.353, *p* = 0.003) and DSS (HR = 1.406, *p* = 0.003) in KIRC (Table 2). Furthermore, the associations between PTX3 and tumor signaling pathway scores were examined in KIRC. Consistent with the GSEA analysis of pan-cancers, Supplementary Figure S5 shows that PTX3 is correlated with diverse tumor signaling pathways such as “inflammatory response”, “IL-10 anti-inflammatory signaling”, “TGF-β”, “ferroptosis”, and “apoptosis”, whereas, a more real mechanism of PTX3 in KIRC remains to be explored in the future.

**Figure 10:**
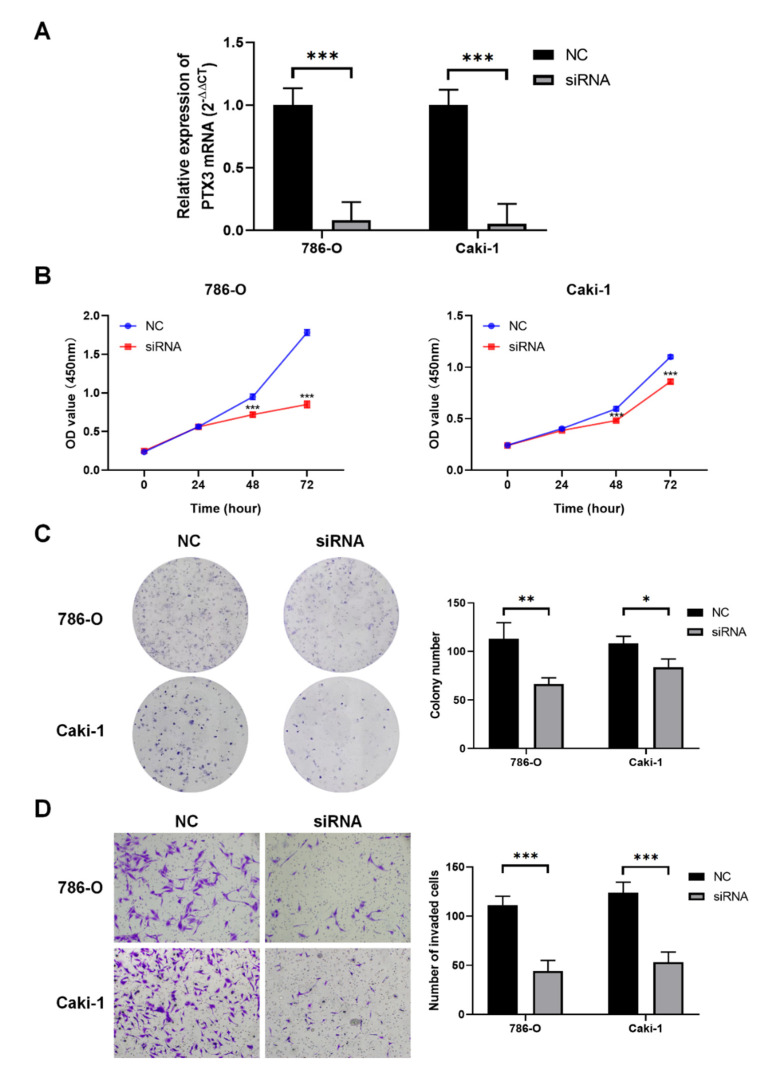
PTX3 function in KIRC: (**A**) The interference efficiency of PTX3 siRNA was evaluated by qRT-PCR; (**B**,**C**) KIRC cells’ proliferation was determined by CCK-8 (**B**) and colony assays (**C**); (**D**) blocking PTX3 expression could inhibit KIRC cell migration and invasion. (Data are shown as the mean ± SD of three replicates. * *p* < 0.05, ** *p* < 0.01, *** *p* < 0.001 by ANOVA test).

As presented in Figure 10A, successful transfection of the Caki-1 and 786-O cells with PTX3 siRNA was confirmed by the PCR results. According to the CCK-8 and colony assays, blocking PTX3 significantly inhibited the proliferation and growth of KIRC cells (Figure 10B,C). In addition, cell migration and invasion were inhibited by PTX3 siRNA (Figure 10D). Meanwhile, to explore whether PTX3 exerted a direct pro-invasive effect in KIRC, we used the PTX3 expression plasmids, transfectant pCMV6-entry PTX3 open reading frame (ORF) clones, and a negative control plasmid to transfect the 786-O and Caki-1 cells. As a result, PTX3 could induce KIRC cell migration and invasion (Supplementary Figure S6). As a result, PTX3 appears to be an oncogene in KIRC.

## 4. Discussion

To deeply elucidate the roles of PTX3, we performed a comprehensive analysis of multi-omics and multifaceted information on expression distribution and regulation, genomic and epigenetic alterations, clinical phenotypes, and immune infiltration. In this comprehensive study, the clinical and prognostic values of PTX3 in pan-cancers were identified via bioinformatics analyses. The function of PTX3 in KIRC was confirmed via cell experiments. PTX3 might act as the treatment and intervention targets, and might provide new immunotherapy strategies for tumor patients.

Previous studies have concurred that PTX3 played an ambivalent role in different cancer types [21,22,23]. For instance, Infante et al. reported that PTX3 overexpression was observed and was related to shorter OS in LUAD [24]. Matarazzo et al. revealed that PTX3 expression decreased in BLCA and exerted an onco-suppressive effect [25]. In line with these findings, we found that PTX3 was aberrantly expressed and could predict distinct prognoses in different cancers. In our study, elevated PTX3 expression was observed in CHOL, GBM, LGG, PAAD, SARC, SKCM, THYM, TGCT, and UCS. In addition, PTX3 had good prediction accuracy of diagnoses across TCGA cancers. This observation indicated the cancer-regulating abilities of PTX3. With the genetic alteration analysis of PTX3, we observed that PTX3 yielded a significant alteration to a great extent in diverse cancers. Likewise, the mutation of PTX3 can influence the prognosis of patients in several cancers. Therefore, one could assume that the expression and mutation of PTX3 are potential parts of tumor biology.

The underlying mechanisms of PTX3 in cancers have been explored previously, including macrophage infiltration, cytokine production, angiogenesis, and genetic instability [26]. In this study, we found that PTX3 and its interacting genes primarily focused on tumor progression and the immune response, including “complement activation”, “humoral immune response”, “acute inflammatory response”, the “PI3K-AKT signaling pathway”, the “MAPK signaling pathway”, etc. Consistent with our results, PTX3 was identified as a possible common mediator between systemic inflammation and cancer [27]. Wang et al. revealed that PTX3 regulated the progression of several tumors through the PI3K/AKT/mTOR pathway [28]. In addition, Cui et al. uncovered that overexpression of PTX3 could inhibit STAD progression mediated by TNF-α, an imperative cytokine member convoluted in the immune system; inflammation; as well as host defence [29]. Concurrently, our GSEA results implied that PTX3 engaged in tumor immune and metabolism pathways such as the “inflammatory response”, “complement”, “KRAS signaling”, “arginine and proline metabolism”, and “Toll-like receptor signaling pathway”. 

Notably, plasma levels of PTX3 were not always in agreement with TCGA data. For instance, PTX3 was silenced in COAD and LIHC across the TCGA data, but PTX3 plasma levels increased [30,31]. In COAD, the authors explained that the PTX3 gene was silenced by DNA methylation of the promoter and a putative enhancer, while increased PTX3 plasma levels reasonably reflected cancer-related inflammation associated with tumor growth [6]. Similarly, Zhang et al. found that increased plasma levels of PTX3 were associated with poor prognoses of COAD patients [32]. In addition, in LIHC patients, polymorphisms may result in higher PTX3 plasma levels [31]. At present, the underlying mechanisms of plasma PTX3 levels were not in agreement with TCGA data in tumors were not fully elucidated. Some possible explanations were listed as follows: (1) PTX3 reflected the inflammation level in the body, and cancer-related inflammation resulted in elevated plasma levels of PTX3. (2) The expression of PTX3 is under the control of epigenetic regulation. PTX3 expression was epigenetically regulated in selected human tumors (e.g., leiomyosarcomas, SKCM, and CRC) by methylation of the promoter region and of a putative enhancer [6]. Rubino et al. showed that two enhancers differently regulated PTX3 expression, which emerged as important fine regulators of PTX3 expression in inflammation and cancer [30]. We also explored the correlation of PTX3 expression with DNA methylation and conducted a KM survival analysis. We found that PTX3 expression was negatively correlated with promoter methylation levels and PTX3 promoter methylation could influence cancer patient prognosis.

In this study, our initial exploration showed that PTX3 was related to ICIs, as well as TMB, MSI, and NEO s cores, which might predict immunotherapy efficacy for cancers. Remarkably, we disclosed that PTX3 was inversely related to ICIs in TGCT, while PTX3 was also negatively correlated with TMB, MSI, and NEO scores in CESC, STAD, and UCEC, indicating the possible resistance to immunotherapy in these cancers. Since ICI therapy is a promising treatment for human tumors, it becomes rather necessary to explore additional promising treatments that enhance and collaborate with ICI therapy [33,34,35]. Cancer researchers have identified TMB, MSI, and NEO as immune-related therapeutic targets [36,37]. Emerging evidence has confirmed that ICI blockade therapy is more effective when there is a high TMB and NEO, since higher TMB and NEO expose more tumor neoantigens [38,39]. In addition, a good response to immunotherapy has been observed for MSI patients with DNA mismatch repair deficiency [40]. Briefly, our current research delineates the relationships between PTX3 and immunotherapy strategies, which could be instructive for clinicians. Emerging evidence has demonstrated that PTX3 facilitated cellular proliferation and conferred resistance to apoptosis by altering cell cycle signaling, thereby, promoting tumor escape from immunosurveillance [27], in support of our observation. Notwithstanding, since PTX3 plays a complex and even contradictory role in immunology, further studies are needed.

Lastly, we validated the function of PTX3 in KIRC. As urologists, we focused on urologic neoplasms, mainly including BLCA, PRAD, and KIRC. Regarding BLCA and PRAD, the bioinformatics analysis did not show statistical significance. However, we noted that PTX3 showed significant overexpression and predicted poor survival in KIRC with the bioinformatics analysis; therefore, we assumed that PTX3 might function in KIRC. Despite the advancements in cross-sectional medical imaging technology, the biomarker of KIRC is still lacking. Thus, we preliminarily explored PTX3 oncogenic function in KIRC with cell experiments. Netti et al. demonstrated that PTX3 could modulate the immunoflogosis in TME via activating the classical pathway of the complement system, which, in turn, led to lower survival rates in KIRC patients [12]. Similar to their study, we identified the oncogenic role of PTX3 and clarified that PTX3 was an independent prognosis factor in KIRC. Specifically, KIRC cancer cell growth and migration could be significantly suppressed by blocking PTX3 with siRNA in vitro. In the future, we would try to confirm PTX3 function in vivo and in vitro via blocking PTX3 with a monoclonal antibody or therapeutic antibody. 

We systematically conducted a pan-cancer analysis of PTX3, which might be clinically valuable for precise and personalized treatment. First, we focused on two hotspots, the COVID-19 biomarker PTX3 and immunotherapy, synthetically underscoring their importance in human cancers. In the meantime, we determined that tumor patients may benefit from immunotherapy as PTX3 correlated with immunotherapy predictors. Lastly, cell assays were conducted to confirm the carcinogenic role of PTX3 in KIRC. This study, however, has limitations. Our study derives mainly from the computational analysis of genomic data and in vivo and in vitro tests are needed to determine the exact mechanisms of PTX3 in cancers. In addition our study does have a limitation on the connection of PTX3 with immunotherapy. The role of PTX3 in immunotherapy should be further validated in clinical and in cell experiments such as coculturing tumor cells with immune cells using the interference for PTX3. 

## 5. Conclusions

In conclusion, PTX3 expression was associated with tumor survival and immunotherapy response, suggesting that PTX3 might serve as a biomarker and a reference for predicting the efficacy of immunotherapy. Tumor patients with high PTX3 expression seem to confer a worse prognosis and their immunotherapy requires careful and wise choices against different cancers.

## Figures and Tables

**Table 1:** Correlations between PTX3 expression and clinicopathological characteristics in TCGA-KIRC.

Characteristics	Odds Ratio (OR)	*p*-Value
Age (>60 vs. ≤60)	0.674 (0.479–0.946)	0.023 *
Gender (female vs. male)	1.097 (0.769–1.566)	0.608
T stage (T3 and T4 vs. T1 and T2)	1.249 (0.877–1.781)	0.219
N stage (N1 vs. N0)	3.342 (1.128–12.220)	0.041 *
M stage (M1 vs. M0)	1.497 (0.922–2.453)	0.105
Pathologic stage (Stage III and Stage IV vs. Stage I and Stage II)	1.061 (0.749–1.504)	0.738
Histologic grade (G3 and G4 vs. G1 and G2)	0.962 (0.684–1.354)	0.826

* p < 0.05.

**Table 2:** Multivariable Cox regression of PTX3 and clinical features of KIRC in TCGA.

Characteristics	OS		DSS	
	Hazard Ratio	*p*-Value	Hazard Ratio	*p*-Value
Age (>60/≤60)	1.673 (1.090–2.569)	0.019 *	--	--
Gender (male/female)	--	--	--	--
T stage (T3 and T4/T1 and T2)	1.094 (0.460–2.599)	0.839	0.865 (0.354–2.114)	0.751
N stage (N1 and N2 and N3/N0)	1.131 (0.528–2.420)	0.751	0.873 (0.364–2.091)	0.760
M stage (M1/M0)	2.303 (1.340–3.960)	0.003 **	2.977 (1.627–5.448)	<0.001 ***
Pathologic stage (Stage III andIV/Stage I and II)	1.638 (0.634–4.236)	0.308	4.043 (1.319–12.393)	0.015 *
Histologic grade (high grade/low grade)	1.671 (1.015–2.751)	0.043 *	1.977 (1.013–3.857)	**0.0468**
PTX3 expression (high expression/low expression)	1.353 (1.111–1.648)	0.003 **	1.406 (1.125–1.757)	0.003 **

* p < 0.05, ** *p* < 0.01, *** *p* < 0.001.

## Data Availability

Data presented in the paper are available upon request from the corresponding author.

## Supplementary Materials

The following supporting information can be downloaded at: www.mdpi.com/article/10.3390/cancers14184438/s1. Supplementary Table S1: Main reagents and consumables mentioned in the text, Supplementary Figure S1: The diagnosis prediction accuracy of PTX3 in pan-cancer by ROC analysis, Supplementary Figure S2: Mutation of PTX3. (A) PTX3 genetic alteration type and frequency in pan-cancer. (B) The mutation counts and types of PTX3. (C) The general mutation sites and number of PTX3, Supplementary Figure S3: A summary of survival between PTX3 CNV and wide type, Supplementary Figure S4: GSEA of PTX3 in pan-cancer. The pan-cancer functional Hallmark (A) and KEGG (B) terms of the PTX3, Supplementary Figure S5: Correlation of PTX3 with tumor signaling pathways in KIRC, Supplementary Figure S6: PTX3 function in KIRC. (A) The transfected efficiency of PTX3 expression plasmid and negative control was evaluated by qRT-PCR. (B) PTX3 overexpression could induce KIRC cell migration and invasion.

## Abbreviations

ACC, acute myeloid leukemia; BLCA, bladder urothelial carcinoma; BRCA, breast invasive carcinoma; CESC, cervical squamous cell carcinoma and endocervical adenocarcinoma; CHOL, cholangiocarcinoma; CI, confidence interval; COAD, colon adenocarcinoma; CNV, copy number variation; DSS, disease specific survival; ESCA, esophageal carcinoma; GBM, glioblastoma multiforme; GEO, Group on Earth Observations; GSEA, gene set enrichment analysis; HNSC, head and neck squamous cell carcinoma; HR, hazard ratio; KICH, kidney chromophobe; KIRC, kidney renal clear cell carcinoma; KIRP, kidney renal papillary cell carcinoma; LAML, acute myeloid leukemia; LIHC, liver hepatocellular carcinoma; LGG, lower grade glioma; LUAD, lung adenocarcinoma; LUSC, lung squamous cell carcinoma; MESO, mesothelioma; OS, overall survival; OV, ovarian serous cystadenocarcinoma; PAAD, pancreatic adenocarcinoma; PCPG, pheochromocytoma and paraganglioma; PRAD, prostate adenocarcinoma; READ, rectum adenocarcinoma; SARC, sarcoma; SKCM, skin cutaneous melanoma; STAD, stomach adenocarcinoma; TCGA, The Cancer Genome Atlas; TGCT, testicular germ cell tumor; THYM, thymoma; THCA, thyroid carcinoma; TME, tumor microenvironment; UCS, uterine carcinosarcoma; UCEC, uterine corpus endometrial carcinoma; UVM, uveal melanoma.
