# Supplementary material for: Pan-Cancer Analysis of Pentraxin 3: A Potential Biomarker of COVID-19
Source: Cancers (Basel). 2022 Sep 13;14(18):4438. doi: 10.3390/cancers14184438 (PMC9496739; doi:10.3390/cancers14184438)
Supplement: Supplementary file 1 [file cancers-14-04438-s001.zip › cancers-1876072-supplementary.pdf]

-

SUPPLEMENTARY MATERIAL

catalogue

SUPPLEMENTARY MATERIAL........1
1. Supplementary Table S1........2
2. Supplementary Figures........3
2.1 **Supplementary Figure S1**........3
2.2 **Supplementary Figure S2**........4
2.3 **Supplementary Figure S3**........5
2.4 **Supplementary Figure S4**........6
2.5 **Supplementary Figure S5**........7
2.6 **Supplementary Figure S6**........8

1. **Supplementary Tables**

1.1. Supplemental Table S1. Main reagents and consumable used in this article.

Reagents	Vendor	Catalogue Number	Information
Primer-PTX3	Tsingke Biotechnology	/	Forward: 5’-AGGCTTGAGTCTTTTAGTGCC-3’<br>Reverse: 5’-ATGGATTCCTCTTTGTGCCATAG-3’
Primer-Actin	Tsingke Biotechnology	/	Forward: 5’- ATGACTTAGTTGCGTTACACC -3’<br>Reverse: 5’- GACTTCCTGTAACAACGCATC -3
siRNA-PTX3	HIPPOBIO	202205050057	**siRNA-NC** Sence: UUCUCCGAACGUGUCACGUTT<br>Anti-Sence: ACGUGACACGUUCGGAGAATT<br>**siRNA-PTX3** Sence: CCACAUGGAGGAGCUCAGUAUTT<br>Anti-Sence: AUACUGAGCUCCUCCAUGUGGTT
Lipofectamine 3000	Invitrogen	PH0429	Carlsbad, CA, USA

-

2. Supplementary Figures

2.1 **Supplementary Figure S1**

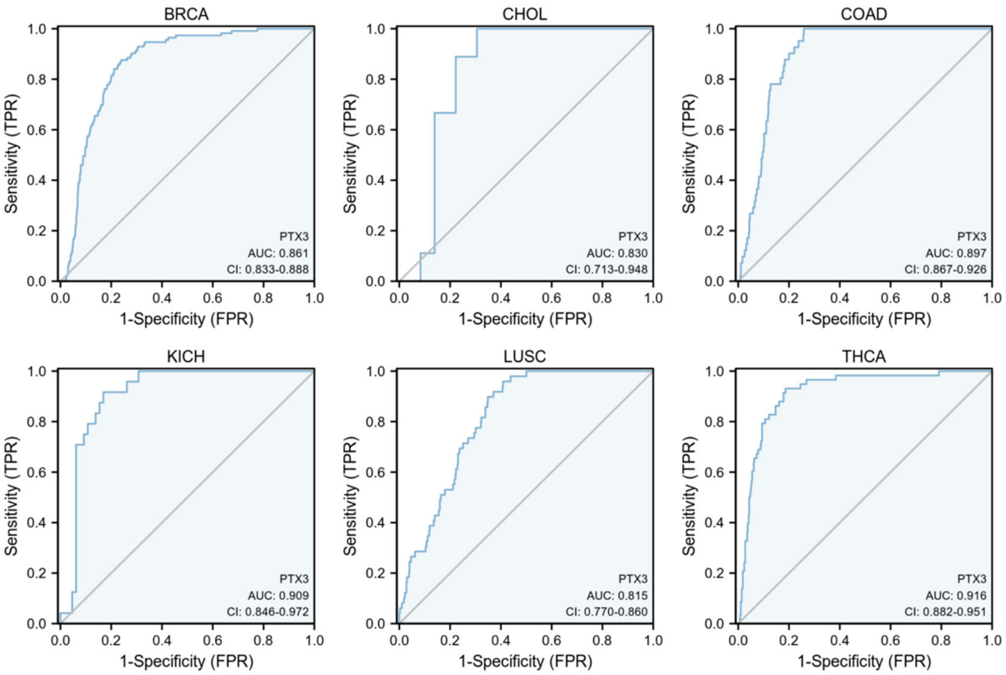

Supplementary Figure S1丨 The diagnosis prediction accuracy of PTX3 in pan-cancer by ROC analysis.

2.2 Supplementary Figure S2

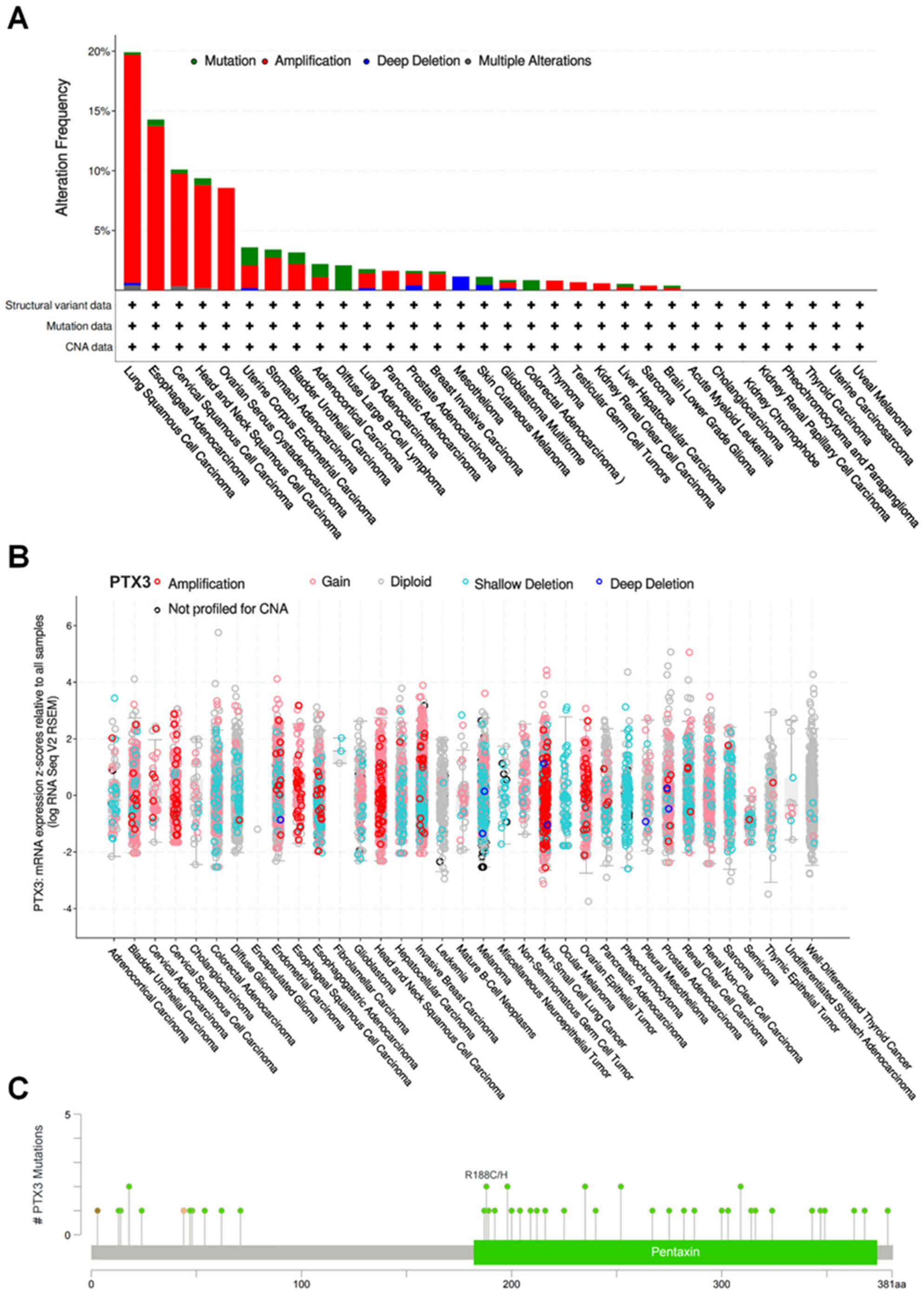

Supplementary Figure S2 | Mutation of PTX3. (A) PTX3 genetic alteration type and frequency in pan-cancer. (B) The mutation counts and types of PTX3. (C) The general mutation sites and number of PTX3.

-

2.3 **Supplementary Figure S3**

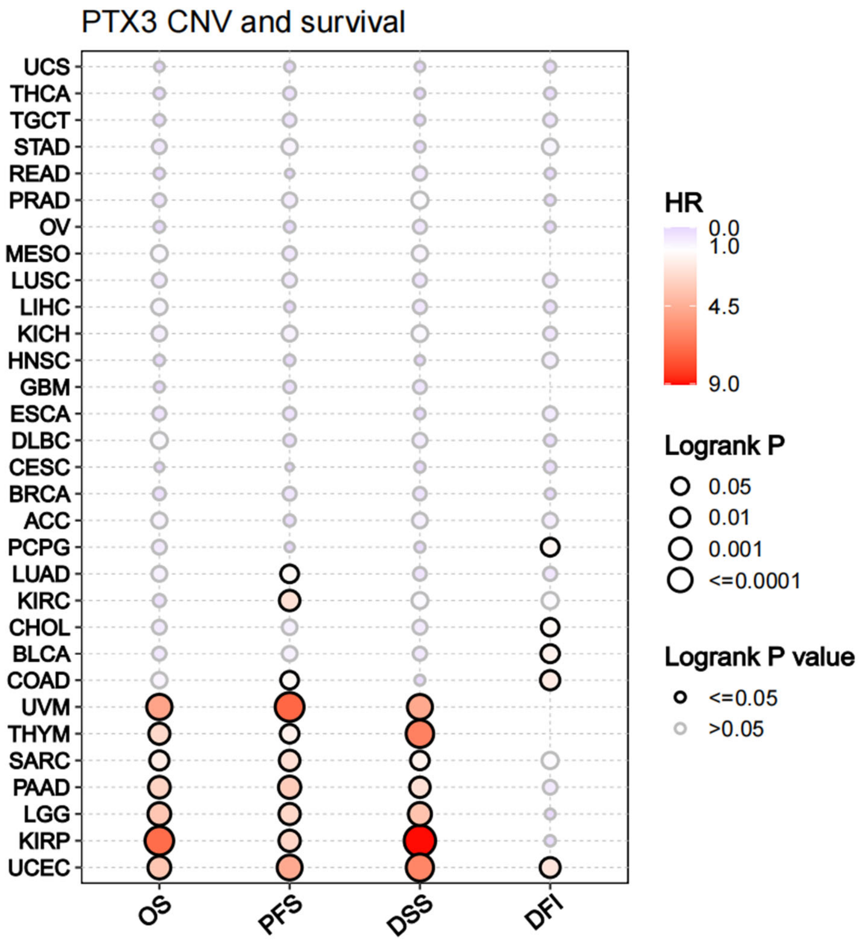

Supplementary Figure S3│ A summary of survival between PTX3 CNV and wide type.

2.4 **Supplementary Figure S4**

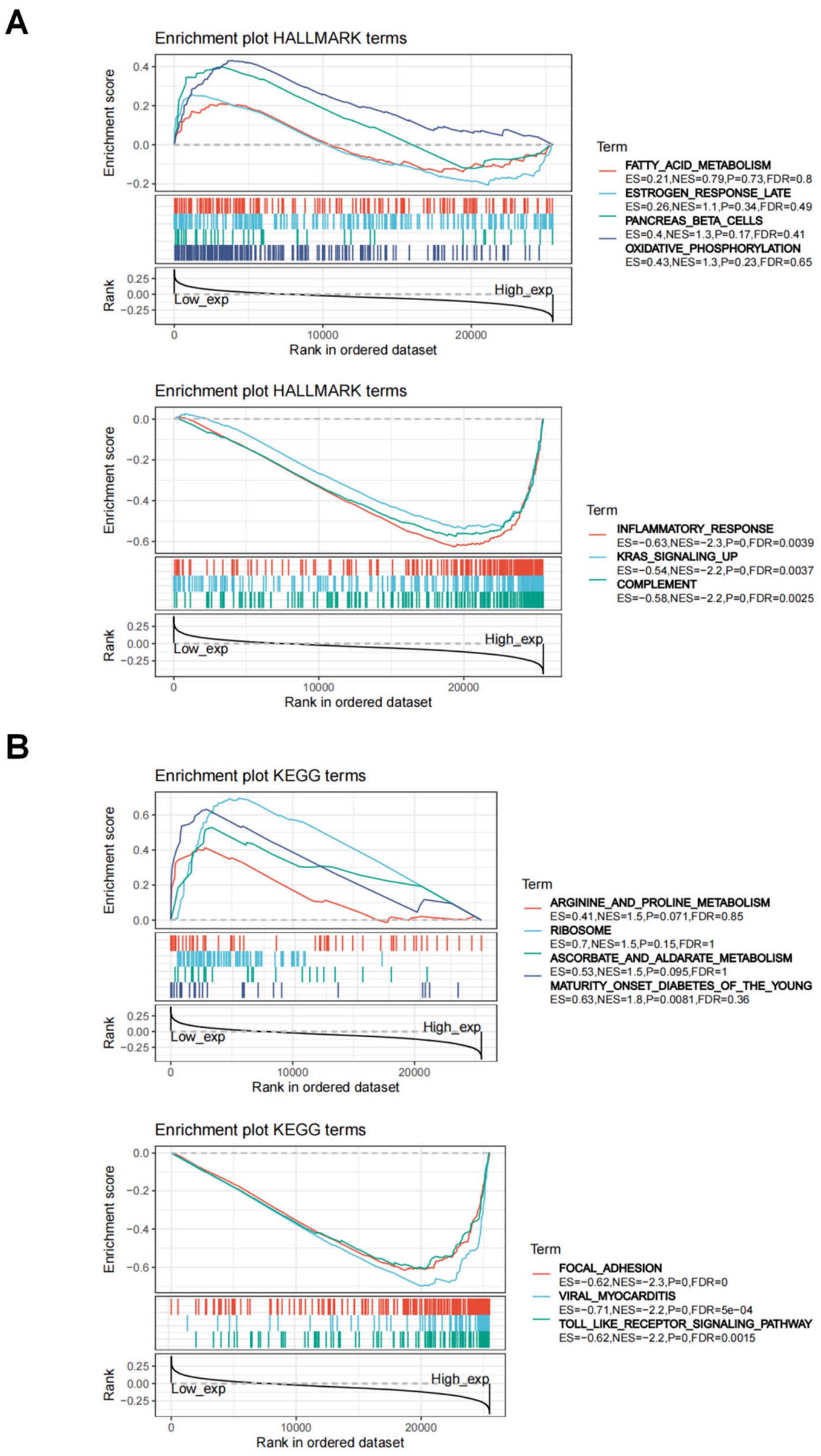

Supplementary Figure S4 | GSEA of PTX3 in pan-cancer. The pan-cancer functional HALLMARK (a) and KEGG (b) terms of the PTX3.

2.5 Supplementary Figure S5

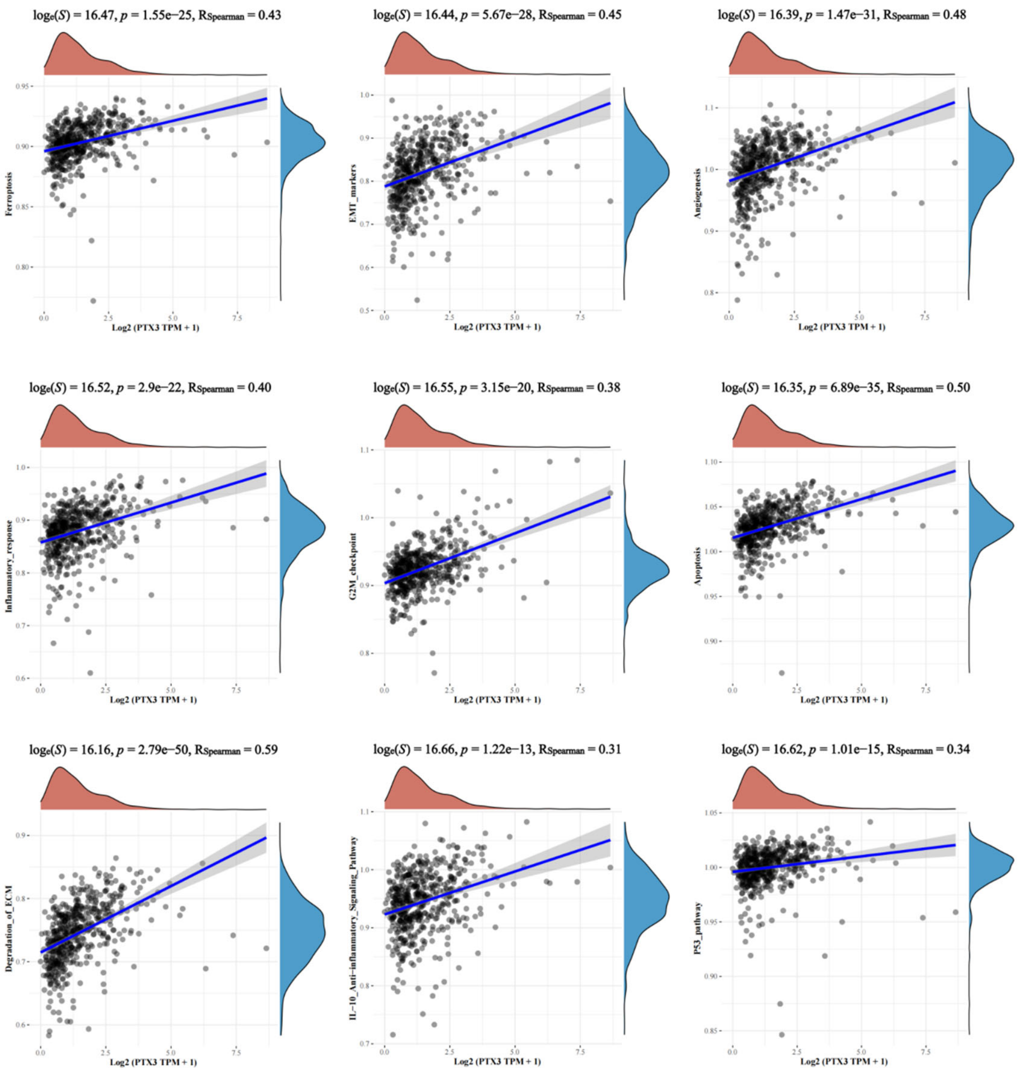

Supplementary Figure S5│ Correlation of PTX3 with tumor signaling pathways in KIRC.

2.6 **Supplementary Figure S6**

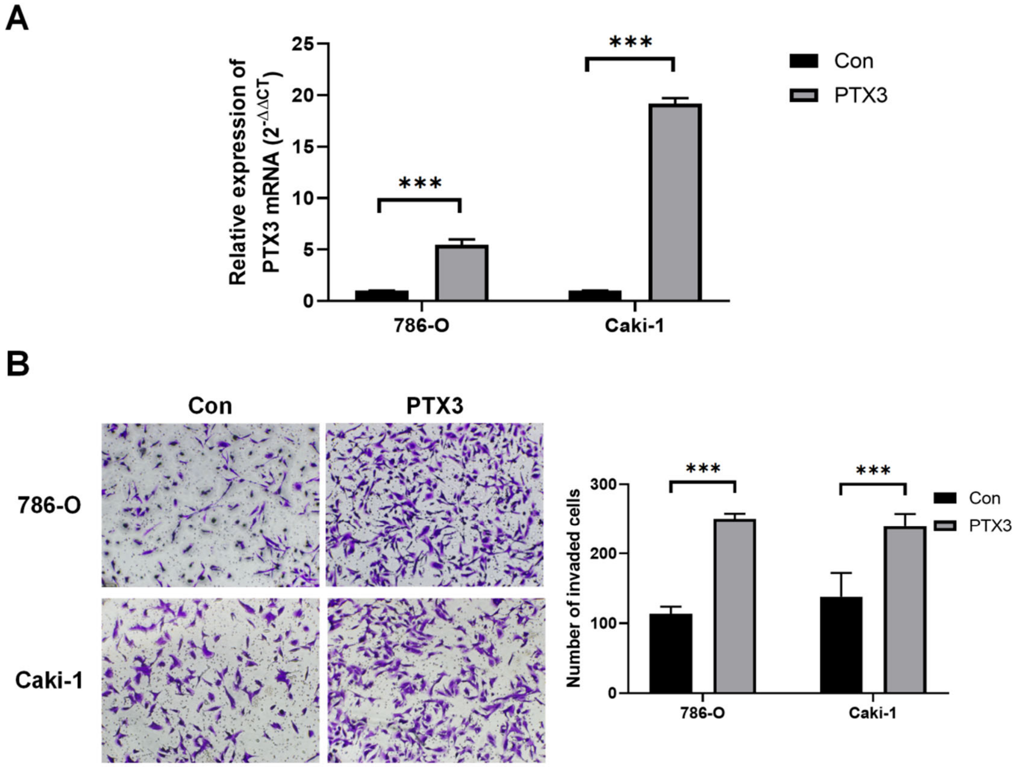

Supplementary Figure S6 | PTX3 function in KIRC. (A) The transfected efficiency of PTX3 expression plasmid and negative control was evaluated by qRT-PCR. (B) PTX3 overexpression could induce KIRC cell migration and invasion. (Data are shown as the mean ± SD of three replicates. * P < 0.05; ** P < 0.01; *** P < 0.001 by ANOVA test).
